# An assessment of catalytic residue 3D ensembles for the prediction of enzyme function

**DOI:** 10.1186/s12859-015-0807-6

**Published:** 2015-11-04

**Authors:** Clemens Žváček, Gerald Friedrichs, Leonhard Heizinger, Rainer Merkl

**Affiliations:** 1 Institute of Biophysics and Physical Biochemistry, University of Regensburg, D-93040 Regensburg, Germany; 2 Faculty of Mathematics and Computer Science, University of Hagen, D-58084 Hagen, Germany

**Keywords:** Catalytic site, Pose comparison, Enzyme function, Enzyme classification

## Abstract

**Background:**

The central element of each enzyme is the catalytic site, which commonly catalyzes a single biochemical reaction with high specificity. It was unclear to us how often sites that catalyze the same or highly similar reactions evolved on different, i. e. non-homologous protein folds and how similar their 3D poses are. Both similarities are key criteria for assessing the usability of pose comparison for function prediction.

**Results:**

We have analyzed the SCOP database on the superfamily level in order to estimate the number of non-homologous enzymes possessing the same function according to their EC number. 89 % of the 873 substrate-specific functions (four digit EC number) assigned to mono-functional, single-domain enzymes were only found in one superfamily. For a reaction-specific grouping (three digit EC number), this value dropped to 35 %, indicating that in approximately 65 % of all enzymes the same function evolved in two or more non-homologous proteins.

For these isofunctional enzymes, structural similarity of the catalytic sites may help to predict function, because neither high sequence similarity nor identical folds are required for a comparison. To assess the specificity of catalytic 3D poses, we compiled the redundancy-free set ENZ_SITES, which comprises 695 sites, whose composition and function are well-defined. We compared their poses with the help of the program Superpose3D and determined classification performance. If the sites were from different superfamilies, the number of true and false positive predictions was similarly high, both for a coarse and a detailed grouping of enzyme function. Moreover, classification performance did not improve drastically, if we additionally used homologous sites to predict function.

**Conclusions:**

For a large number of enzymatic functions, dissimilar sites evolved that catalyze the same reaction and it is the individual substrate that determines the arrangement of the catalytic site and its local environment. These substrate-specific requirements turn the comparison of catalytic residues into a weak classifier for the prediction of enzyme function.

**Electronic supplementary material:**

The online version of this article (doi:10.1186/s12859-015-0807-6) contains supplementary material, which is available to authorized users.

## Background

Enzymes are the workhorses of all metabolic processes observed in nature, which modify their substrates with high specificity and efficiency. Interestingly, on average no more than 3.5 of the approximately 80 to several hundred residues that constitute an enzyme are directly engaged in catalysis [1]. Commonly, these residues are named catalytic residues and the catalytic residues of one enzyme are termed catalytic site or simply site. In certain enzymes, metal ions, cofactors, or water molecules are also involved in catalysis, but the catalytic site is central to the function of an enzyme.

It is known that evolutionary unrelated proteins can catalyze the same biochemical reactions and these non-homologous isofunctional enzymes have been named NISE [2]. Along these lines, algorithms have been developed that compare the poses, i. e. the relative spatial (3D) orientation of catalytic residues from different enzymes. These comparisons are based on the assumption that highly similar poses of sites are indicative of similar reactions and support the prediction of enzyme function in the absence of a more global sequence or fold similarity.

A pioneering and very flexible algorithm that allows the user to specify a site is PINTS [3]; the alternative GASPS utilizes a machine learning approach and automatically identifies sets of 3 – 10 residues that maximize function prediction [4]. Applied to four protein superfamilies, GASPS was found to perform comparable or better than commonly used methods of annotation transfer like PSI-BLAST [4]. Alternatively, the catalytic site identification server provides users with protein annotations based on structural matches with entries of the PDB [5]. For the more recently introduced algorithm CMASA, which uses a similar approach, the authors reported the detection of 166 putative catalytic sites [6]. Methods like ProFunc [7] or ASSIST [8] combine analysis of protein sequence and structure using several algorithms. The performance of these two approaches is comparable and ASSIST predicted for 34 of 54 randomly chosen enzymes the correct Enzyme Commission (EC) number [8].

With the advent of highly sensitive methods like HHsearch, which is based on a comparison of Hidden Markov Models [9], the sensitivity in detecting homologous proteins has increased drastically: even if the pairwise sequence identity of homologous proteins is no greater than 20 %, HHsearch identifies 50 % of these relationships [9]. Although homologous enzymes may catalyze quite different reactions, such hits narrow down the number of putative functions if similarity is high for the full sequences [10]. Thus, the major challenges of a catalytic site comparison are the cases in which no homologous enzymes allow for an annotation transfer.

Recently, we have implemented CLIPS-4D, which predicts functionally important residues, i. e. catalytic and ligand binding ones [11]. For these predictions, CLIPS-4D only uses features deduced from 3D structure and homologous sequences like solvent accessibility or residue conservation and does not transfer the annotation of known sites. A straightforward utilization of these data would be a functional assignment based on the pose of the putative catalytic residues in order to predict function for unknown enzymes. Hence we had an interest in determining the performance of pose comparison for more difficult cases.

Thus we focused on non-homologous isofunctional enzymes, where function assignment by sequence comparison will fail in most cases. To this end, we estimated the number of NISE, i. e. those cases which are due to convergent evolution. In convergent evolution, non-homologous enzymes evolve in separate biological contexts to catalyze the same or a similar biochemical transformation.

Our analysis of the SCOP database [12] identified 98 out of 873 specific enzyme functions as NISE, which argues in favor of pose comparison for function assignment. However, for a successful application, the predictive power of this approach has to reach an acceptable level. In order to assess classification performance, we i) compiled a representative and redundancy-free data set of catalytic sites and ii) utilized the program Superpose3D [13] to compare their poses. The function of the enzymes was compared based on the assigned EC classes [14] and GO terms [15]. It turned out that for sites consisting of two or more residues, the specificity of pose comparison is relatively low; similar poses were found in enzymes catalyzing completely different reactions. Consequently, this finding makes clear that a pose comparison alone can only help in a few cases to unambiguously assign enzyme function.

## Methods

### ENZ_SITES, a redundancy-free set of catalytic sites

The Catalytic Site Atlas (CSA) is a comprehensive and frequently used resource of catalytic sites [16]. This database consists of two types of annotations: a hand‐annotated set containing information extracted from the primary literature, using well-defined criteria to assign catalytic residues and an additional homologous set, containing annotations inferred by PSI‐BLAST and a sequence alignment to one entry of the manually curated data set [16]. In order to concentrate on highly reliable data, we focused on the manually curated sites, which were in version 2 of CSA from 928 different PDB [17] entries. Additionally, we queried the databases BRENDA [18] and PDBsum [19] (versions as of January 2014) in order to maximize the number of assigned functions, i. e. EC numbers.

For our purposes, we had to exclude a large number of entries: 317 sites consisted of just one residue, which renders the comparison of poses insignificant, as the 3D superposition of the two corresponding side chains will always indicate high similarity. For 34 sites, at least one residue was not a canonical amino acid residue and 126 sites were excluded, as the catalytic residues were located at different chains of the PDB entry, which is a configuration we could not analyze. 41 sites were from proteins without an EC number and 77 sites from multifunctional enzymes; i. e. more than one EC number was listed. 36 sites had no SCOP entry which made it impossible to decide on homology, and 8 sites were removed due to inconsistencies in the CSA data and the corresponding PDB entries. After this filtering process, the data set, which we named ENZ_SITES, consisted of 695 sites. The names of these CSA entries are listed in Additional file 1. The average number of catalytic residues per site was 3.4, which is in agreement with previous findings [1]; the minimum were two (207/695, 29.8 % of all sites) and the maximum ten residues.

### Performance measures

To assess the performance of a classification, we determined the sensitivity

1 $$ \operatorname{Sensitivity}=\frac{\mathrm{TP}}{\mathrm{TP}+\mathrm{F}\mathrm{N}} $$

and the MCC value

2 $$ \mathrm{M}\mathrm{C}\mathrm{C}=\frac{\mathrm{TP}\times \mathrm{T}\mathrm{N}\hbox{-} \mathrm{F}\mathrm{P}\times \mathrm{F}\mathrm{N}}{\sqrt{\left(\mathrm{T}\mathrm{P}+\mathrm{F}\mathrm{N}\right)\left(\mathrm{T}\mathrm{P}+\mathrm{F}\mathrm{P}\right)\left(\mathrm{T}\mathrm{N}+\mathrm{F}\mathrm{P}\right)\left(\mathrm{T}\mathrm{N}+\mathrm{F}\mathrm{N}\right)}} $$

MCC values [20] are considered a fair performance measure as they are deduced from all classified cases. In both formulae, TP is the number of true positives, TN the number of true negatives, FP the number of false positives, and FN the number of false negatives.

### Normalized RMSD values

To normalize for the extent of the sites, we tested two scores, which take into account the number of residues that were matches:

3 $$ RMS{D}_{N1}\left(c{s}_i,c{s}_j\right)=\frac{RMSD\left(c{s}_i,c{s}_j\right)}{\left| ms\right|-1} $$

4 $$ RMS{D}_{N2}\left(c{s}_i,c{s}_j\right)=\frac{RMSD\left(c{s}_i,c{s}_j\right)}{ \max \left(1,\kern0.5em 3\times \left| ms\right|-6\right)} $$

Here, *RMSD*(*cs*_*i*_, *cs*_*j*_) = Superpose3D(*cs*_*i*_, *cs*_*j*_, all-atom mode) is the root mean square deviation (RMSD) value determined for the comparison of sites *cs*_*i*_ and *cs*_*j*_ in all-atom mode of Superpose3D [13]; |*ms*| is the number of matched, i. e. successfully superposed residues. For *RMSD*_*N*2_, the score decreases stronger than with *RMSD*_*N*1_ if *ms* consists of a larger set of residues.

## Results and discussion

### Homology is indicative of substrate specificity, but not of enzymatic function

Based on a detailed analysis of 33 sites, it has been deduced that convergent evolution of catalytic sites is not rare [21]. A more comprehensive analysis resulted in 185 confirmed EC nodes, i. e. enzymatic functions, where different protein folds were present [2]. The Enzyme Commission numbering scheme [14] groups the chemical reactions catalyzed by enzymes in six classes. These are (1) oxidoreductases, (2) transferases, (3) hydrolases, (4) lyases, (5) isomerases, and (6) ligases. Beneath each of the six classes, three levels of subclasses describe the specific enzymatic reaction in more detail. The third subclass level specifies the reaction and the fourth level the specific substrate [22, 23].

We were interested in estimating the number of NISE on the level of the enzymatic reactions and, additionally, on the level of their individual substrates. In order to determine the evolutionary relationship of enzymes, we utilized the hierarchical classification of the SCOP database (release 1.75) [12]. SCOP is manually maintained by experts and its superfamily level is regarded as the most reliable standard for remote homologs [24]. Thus, we considered two enzymes as homolog if they shared the same SCOP superfamily.

**Fig. 1:**
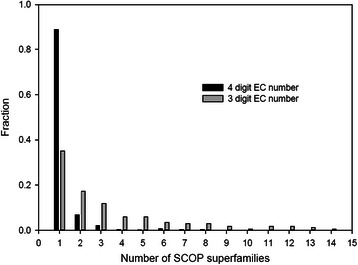
Enzymatic function and their evolution in different SCOP superfamilies. For the dark bars, enzymes were grouped according to their four-digit EC number and the number of SCOP superfamilies was deduced for each of these substrate specific enzymatic functions. The light bars show the histogram deduced from a compilation of three-digit EC numbers, which subsumes enzymes with the same function but different substrates

We identified all single-domain enzymes that had been assigned exactly one full EC number. These 8102 enzymes were grouped according to their EC number, and for each of these 873 enzyme-catalyzed reactions the number of SCOP superfamilies was determined. Results are shown in Fig. 1. The fraction of reactions (four-digit EC number) catalyzed exclusively by homologous enzymes was 89 %. 98 (11 %) of these reactions were found in at least two different superfamilies and no more than 2 % in five or more superfamilies. Most extreme were the non-specific serine/threonine protein kinase (EC 2.7.11.1) that had 19 superfamilies and the endo-1,4-beta-xylanase (EC 3.2.1.8), the protein-tyrosine-phosphatase (EC 3.1.3.48) and the histidine protein kinase (EC 2.7.13.3) that had 8 superfamilies. Note that these values are upper bounds for the occurrence of convergent evolution, as we assumed that enzymes from different superfamilies are non-homologous. This is not true in all cases, *e. g*. the assignment of (βα)_8_-barrels to several SCOP superfamilies is too conservative [25].

According to EC nomenclature, enzymes catalyze the same reaction if the first three digits of their EC number are identical. After applying the same grouping as above to the first three EC digits, the number of functions represented exclusively by homologous enzymes dropped to 35 %, and the number of functions with five or more superfamilies was 30 %. These findings make clear that homology - and consequently sequence similarity - is a strong indicator of a specific substrate, but not of the more general enzymatic function. Taken together, these numbers are in agreement with previous findings, which identified 185 EC nodes with two or more experimentally characterized (or predicted) structurally unrelated proteins [2].

### The all-atom representation and a normalized RMSD are best suited for site comparison

As shown above for many cases, the same enzymatic function can be found in different superfamilies. However, these comparisons do not permit a conclusion on the specific arrangements and similarities of catalytic sites.

We wanted to assess the 3D similarity of these sites based on a representative data set. Unfortunately, it is unclear for many enzymes which residues are directly involved in catalysis. This is why we had to concentrate on a smaller, redundancy-free set of catalytic sites, which we named ENZ_SITES; see Methods. It consists of 695 sites and is based on the Catalytic Site Atlas, which precisely enumerates residues that are directly involved in catalysis [16]. Thus, for all ENZ_SITES entries, the 3D orientation of the corresponding catalytic residues is known as well as the SCOP classification [12]. The function of these enzymes is given by their EC number and alternatively by a set of GO terms [15] that were inferred from the Gene Ontology Annotation Database [26] for 618 ENZ_SITES entries.

**Fig. 2:**
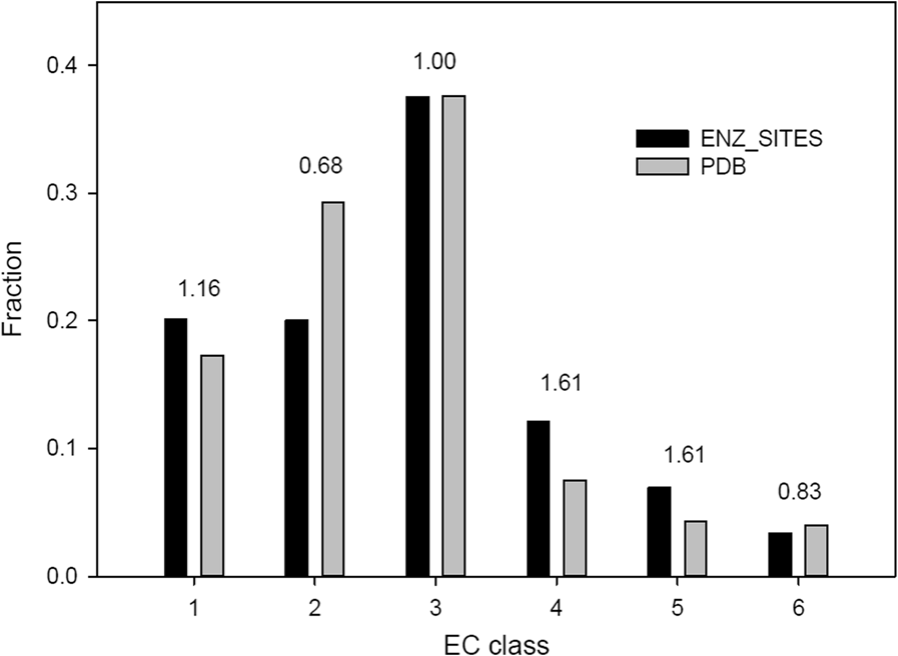
Occurrence of enzymes from the six EC classes. The dark bars give the fraction of the enzymes in ENZ_SITES, the light bars their fraction in the PDB. On top of the bars, the ratio of the corresponding fraction values is printed. The value of 1.16 indicates that enzymes from EC class 1 are 16 % overrepresented in ENZ_SITES with respect to their occurrence in the PDB

To begin with, we identified the most suitable 3D representation of side chains and the type of score that maximizes the number of correct predictions. For a first analysis, we distributed all entries of ENZ_SITES to six sets according to the first digit of the corresponding EC number. Figure 2 confirms that the EC class distribution observed in the PDB is preserved to a great extent in ENZ_SITES and demonstrates that our selection of enzymes is not strongly biased with respect to their function. For the 3D comparison of sites, we chose the program Superpose3D [13], as it offers a large number of ways to represent side chains and amino acid equivalency rules. We altered the source code so that we could enumerate all possible matches up to a certain, predefined RMSD cut-off. These matches varied with respect to |*ms|*, which is the number of superimposed residues. We compared each catalytic site *cs*_*i*_ with all other entries *cs*_*j*_ that belonged to a different SCOP superfamily, in order to compare sites that were most likely from non-homologous enzymes. We counted the prediction as correct (TP, functional match), if the first digit of the EC numbers of *cs*_*i*_ and the most similar site *cs*_*m_s*_ were identical. If *cs*_*m_s*_ was from a different EC class, it was an FN prediction.

To score a comparison of sites, two parameters have to be chosen: these are i) the representation of residues and ii) a measure that assesses the 3D difference of the two sites. Considering residue representation, we alternatively used for each residue the C_α_-atom, the C_α_- plus the C_β_-atom, the C_α_-atom plus the centroid of the side chain, and the all-atom representation. Moreover, we modeled the structural resemblance of tyrosine and phenylalanine and of isoleucine and valine. With respect to the overall similarity of the poses, we compared as a measure the pure RMSD as determined by Superpose3D, two terms (*RMSD*_*N*1_, *RMSD*_*N*2_) aimed at normalizing by the number of matched residues (*ms*, see Methods), and the scores introduced by Stark et al*.* [27] and by Torrance et al*.* [28]. Table 1 lists the resulting sensitivity values, which were computed according to equ. (1). It turned out that the combination of the all-atom representation with strict residue equivalences in combination with the *RMSD*_*N*2_ value gave the highest sensitivity of 0.32. The finding that the all-atom representation is superior to other ones is in agreement with previous studies [29]. Interestingly, normalizing the RMSD value also improved the selection of templates for protein design, as has been demonstrated for the TM-score [30].

**Table 1:** Numbers of TPs and sensitivity for different representations of catalytic sites and for scores to compare their poses

Method	TP	Sensitivity
C_α_	177	0.26
C_α_ + C_β_	206	0.30
C_α_ + centroid	215	0.31
*EQUI*(Y/F, I/L)	216	0.31
Stark score	214	0.31
Torrance score	213	0.31
RMSD	211	0.30
*RMSD*_*N*1_	218	0.31
*RMSD*_*N*2_	220	0.32

In the upper part, the column Method lists the performance reached for different representations of the catalytic sites. C_α_ and C_β_ indicate a representation restricted to the respective atoms, the centroid represents the full sidechain with one point in 3D. For *EQUI*(Y/F, I/L), Superpose3D considers the residue pairs Y, F and I, L as structurally equivalent. The second part of the table lists results deduced by using different scores for the 3D comparison of poses. For these cases, the all-atom mode of Superpose3D was used; for details see text

### Maximizing the MCC as a means of finding optimal cut-off values

So far, we utilized for a classification only the most similar hit *cs*_*m_s*_, independent of the magnitude of the *RMSD*_*N*2_ value. For a classifier that predicts enzyme function, a cut-off value *cu_RMSD*_*N*2_ has to be defined, which decides on the result of the classification: if *RMSD*_*N*2_(*cs*_*i*_, *cs*_*m_s*_) < *cu_RMSD*_*N*2_, then the known function of the best hit with site *cs*_*m_s*_ is transferred to the enzyme with site *cs*_*i*_. In order to find the optimal setting, we used the MCC value, which is considered a fair measure to assess performance on unbalanced sets of positives and negatives, as observed here [31]. More precisely, we searched after the cut-off *cu_RMSD*_*N*2_ that *maximized* the MCC value according to:

5 $$ \begin{array}{l}cu\_RMS{D}_{N2}= \arg \kern0.62em \underset{val}{ \max}\left(MCC\left(RMS{D}_{N2}\left(c{s}_i,c{s}_j\right), val\right)\right)\\ {}\forall c{s}_i,c{s}_j\in \mathrm{E}\mathrm{N}\mathrm{Z}\_\mathrm{SITES}\vee Ident\_ SupFam\left(c{s}_i,c{s}_j\right)=\mathrm{F}\end{array} $$

*Ident_SupFam*(.) was false (F) if the enzymes represented by sites *cs*_*i*_, *cs*_*j*_ belonged to different SCOP superfamilies. Thus, we only compared sites which were most likely from non-homologous enzymes, and we named these comparisons DIFF_SF. For the computation of the MCC values, we only considered the best hit *cs*_*m_s*_. If *RMSD*_*N*2_(*cs*_*i*_, *cs*_*m_s*_) < *val*, then the prediction was counted as TP if the EC classes of *cs*_*i*_ and *cs*_*m_s*_ were identical; otherwise it was a FP prediction. Accordingly, the cases with *RMSD*_*N*2_(*cs*_*i*_, *cs*_*m_s*_) ≥ *val* were considered: if the EC classes of *cs*_*i*_ and *cs*_*m_s*_ were identical, it was a FN, otherwise a TN prediction. The largest MCC value we found was 0.19 for *cu_RMSD*_*N*2_ = 0.65 Å; see Table 2. With these optimal settings, a relatively low number of 61 TP and a comparatively high number of 58 FP cases were generated together with 159 FN and 417 TN predictions.

**Table 2:** Classification performance for pose comparison

	Method	MCC	TP	FP	TN	FN
DIFF_SF	*cu_ RMSD*_*N*2_ = 0.65 Å, 6 EC classes	0.19	61	58	417	159
	*cu_ RMSD*_*N*2_ = 0.21 Å, *cu_ S*_*GO*_ = 0.88, CC, BP, MF	0.44	2	8	608	0
	*cu_ RMSD*_*N*2_ = 0.21 Å, *cu_ S*_*GO*_ = 0.88, BP, MF	0.64	5	5	607	1
	*cu_ RMSD*_*N*2_ = 0.50 Å, *cu_ S*_*GO*_ = 0.75, BP, MF	0.36	35	38	493	52
	*cu_ RMSD*_*N*2_ = 0.21 Å, *cu_ S*_*GO*_ = 0.75, BP, MF	0.29	5	5	587	21
	*cu_ RMSD*_*N*2_ = 0.91 Å, *cu_ S*_*GO*_ = 0.88, BP, MF	0.11	6	279	333	0
	*cu_ RMSD*_*N*2_ = 0.91 Å, *cu_ S*_*GO*_ = 0.75, BP, MF	0.13	20	265	327	6
ALL_ENZ_SITES	*cu_ RMSD*_*N*2_ = 0.37 Å, *cu_ S*_*GO*_ = 0.90, BP, MF	0.29	14	41	546	17
	*cu_ RMSD*_*N*2_ = 0.99 Å, *cu_ S*_*GO*_ = 0.77, BP, MF	0.47	59	78	459	22
	*cu_ RMSD* = 0.85 Å, *cu_ S*_*GO*_ = 0.70, MF	0.57	167	73	325	53
	*best of 10 cu_ RMSD*_*N*2_ = 1.10 Å, *cu_ S*_*GO*_ = 0.75, BP, MF	0.58	163	65	337	53

In all cases, the cut-offs for the comparison of poses (*cu_ RMSD* or *cu_ RMSD*_*N*2_) and sets of GO terms (*S*_*GO*_) are given under Method. The terms CC, BP, and MF indicate which combination of terms from the annotation domains were analyzed, respectively. The columns on the right give the MCC value and the number of TP, FP, TN, and FN cases, which resulted from a classification using these cut-offs. For the experiments labeled DIFF_SF, only poses for enzymes from different superfamilies were compared; for ALL_ENZ_SITES, sites from all entries of ENZ_SITES were compared

### Classifying function based on site comparison and GO terms

Grouping all enzymes into no more than six classes, as we have done so far, is a relatively crude approach that may deteriorate classification performance. The low sensitivity of 0.32 (Table 1) indicates that the number of FN is approximately 2 × TP. This means that highly similar sites possess dissimilar functions to be found in enzymes belonging to different EC classes. To be more specific, we applied a classification scheme with a higher resolution of enzyme function. However, instead of using EC subclasses, we chose the gene ontology, which also allows a precise specification of enzyme activity [15]. The major advantage of an ontology is the possibility of comparing terms by means of a similarity score, which is not feasible for EC subclasses.

The gene ontology describes gene products with respect to three annotation domains: these are the cellular component (CC), where the protein is active, the biological process (BP) the protein is involved in, and the molecular function (MF), which specifies the catalyzed reaction for enzymes. The set of GO terms is fixed and an acyclic graph defines the relationships between terms, which makes possible the comparison of terms and term sets.

For this comparison, several algorithms have been implemented, and we opted for a method proposed recently [32]. This approach computes similarity scores, which are independent of the occurrence of the GO terms in the data set under study. The output is a normalized score *S*_*GO*_(*G*_*i*_, *G*_*j*_) from the range [0, 1], and higher values indicate that the sets *G*_*i*_ and *G*_*j*_ of GO terms are similar. Here, larger values signal that the corresponding enzymes *e*_*i*_ and *e*_*j*_ represented by sites *cs*_*i*_ and *cs*_*j*_ have similar function. We conjectured that GO terms related to the domain CC do not contribute to a classification of enzyme function, therefore we tested several combinations of the three annotation domains. However, the optimal cut-off *cu_S*_*GO*_ was unclear, as the authors did not recommend a default value. This is why we performed a grid search and varied in a systematic manner both *cu_RMSD*_*N*2_ and *cu_S*_*GO*_ in order to maximize the MCC value analogously to the above-mentioned approach. Table 2 summarizes the results; the largest MCC value of 0.64 resulted from an analysis of BP and MF terms and the cut-offs *cu_RMSD*_*N*2_ = 0.21 Å and *cu_S*_*GO*_ = 0.88. The number of only five TP indicated a low classification success. For the least stringent cut-off values which we studied, namely *cu_RMSD*_*N*2_ = 0.91 Å and *cu_S*_*GO*_ = 0.75, the MCC value dropped to 0.13 and the number of FP increased to 265. For the intermediate cut-offs *cu_RMSD*_*N*2_ = 0.50 Å and *cu_S*_*GO*_ = 0.75, the number of FPs was 38, but the MCC value was not higher than 0.36. For a fixed *cu_RMSD*_*N*2_ value, the MCC value increased with a decrease of *cu_S*_*GO*_, as more cases were considered TP; compare the listed results for *cu_RMSD*_*N*2_ = 0.91. As expected, adding GO terms from the CC domain to the functional assignment deteriorated classification performance: the maximal MCC value dropped from 0.64 to 0.44.

### Adding poses of homologs does not improve classification performance

The above results indicate that pose comparison of non-homologous enzymes identifies only a small number of functionally similar enzymes. On the other hand, large protein families are known to exist, which consist of functional diverse enzymes [33]. This is why we also wanted to assess classification performance resulting from a comparison of all ENZ_SITES, and we named these analyses (which also included homologous sites) ALL_ENZ_SITES. Again, the maximal MCC value was determined by means of a grid-search, and results are listed in Table 2. Using the same parameters as above (*RMSD*_*N*2_ and BP, MF), the MCC value was 0.47. As expected, the number of TP increased to 59, but the number of FP rose simultaneously to 78. A classification based on the pure RMSD and GO terms from MF resulted in the MCC value of 0.57. In this case, TP was 167, FP 73 and sensitivity 0.76. It seems that this MCC value is the upper limit of the classification performance: even if we picked the enzyme with the highest *S*_*GO*_-score out of the 10 most similar poses, the maximal MCC value was not higher than 0.58 and the number of TP, FP, TN, and FN did not change notably; see the numbers given in Table 2. For the more stringent cut-offs *cu_RMSD*_*N*2_ = 0.37 Å and *cu_S*_*GO*_ = 0.90, the MCC value was only 0.29.

Are our findings consisting of an optimal MCC value of 0.57 and a sensitivity of 0.76 in agreement with previous results? For their assessment, we selected two recently introduced alternatives, which are the catalytic site identification server [5] and the CMASA algorithm [6]. Both are based on similar methods for the 3D comparison of catalytic sites as introduced above and have been evaluated on larger data sets. For the catalytic site identification server, an MCC value of 0.55 and a sensitivity of 0.85 have been reported [5]. For the CMASA algorithm, a mean MCC value of 0.90 and a sensitivity of 0.86 have been determined. Note that in both assessments, only sites consisting of three or more catalytic residues have been analyzed. Moreover, for the CMASA data set, all three residue sites containing two glycines were eliminated as well. Thus, the lower performance we determined for the 695 entries of our data set ENZ_SITES is most likely due to the additional assessment of the more difficult cases, which are the 207 sites consisting of only two residues. In summary, these findings make clear that pose comparison alone is not sufficient to unambiguously determine enzyme function, if sites consisting of not more than two residues have to be analyzed. Additionally, a pose comparison is not possible for approximately a third of the CSA sites, which consist of only one catalytic residue. These cases were masked out in all of the above mentioned studies.

### Nature’s preference of a limited number of sites complicates function prediction

Our survey of 873 substrate-specific enzymatic functions made clear that approximately 11 % of them have evolved on two or more non-homologous protein structures. On the other hand, the performance of pose comparison was poor both for homologous and especially for non-homologous structures. For the relatively strict cut-offs *cu_RMSD*_*N*2_ = 0.21 Å and *cu_S*_*GO*_ = 0.88, the number of five FP predictions equals the number of five TPs, which indicates that the same residues - arranged in a highly similar orientation - catalyze different reactions. For the other settings tested here, the number of FP was as well similar to those of TP predictions. Why is this the case?

It is known that only the 11 polar and charged residues are generally observed as catalytic residues [34]. Moreover, the combination of residues which occur in catalytic sites is strictly limited: no more than nine residue combinations were found that are repeatedly in use in different and unrelated enzymes [34], which makes the above findings plausible.

On the other hand, an unbiased analysis of enzymes suggested that less than 30 % of the BLAST pair fragments determined above 50 % sequence identity have an identical function [10]. A survey of structurally characterized superfamilies demonstrated that almost 40 % are functionally diverse, i. e*.* different members catalyze reactions with different EC numbers [35]. Thus, a simple annotation transfer by sequence homology is often insufficient to assign function. A striking example is the enolase superfamily, which consists of more than 8000 members that catalyze more than 20 different reactions [36].

How can one assign function in these cases? Most helpful is a combination of orthogonal methods. Among the *in silico* approaches used so far are ligand docking, the analysis of the genomic context, and sequence similarity networks [36]. However, *in silico* ligand docking is not always successful, as the binding of a ligand often induces structural changes and the genomic context is only conserved for microorganisms. Moreover, these methods require a fine-tuning of parameters and their concerted interpretation is difficult. Thus, the assignment of enzyme function still requires human expertise, and the design of reliable classifiers for enzyme function is still an open problem, as has been shown by the CAFA contest [37]. The challenge is to find the most suitable combination of weak classifiers like CLIPS-4D and pose comparison and the integration of disparate data sources in order to form a robust and reliable system for functional assignment.

## Conclusions

For a large number of enzymatic functions, dissimilar sites evolved that catalyze the same reaction and it is the individual substrate that determines the arrangement of the catalytic site and the adjacent residues. It follows that these substrate-specific requirements turn the comparison of catalytic sites into a weak classifier for the prediction of enzyme function: If a site consists of not more than two or three catalytic residues, the specificity of pose comparison is relatively low. Thus, the composition and the 3D arrangement of the site are in most cases not specific for a distinct enzyme function.

## Additional file

Additional file 1:
**Composition of data set ENZ_SITES.** (PDF 84 kb)
