# Supplementary material for: An assessment of catalytic residue 3D ensembles for the prediction of enzyme function
Source: BMC Bioinformatics. 2015 Nov 4;16:359. doi: 10.1186/s12859-015-0807-6 (PMC4634577; doi:10.1186/s12859-015-0807-6)
Supplement: Additional file 1: — Composition of data set ENZ_SITES. (PDF 84 kb) [file 12859_2015_807_MOESM1_ESM.pdf]

Additional file1: The composition of the dataset ENZ_SITES.

For each site, the PDB-ID and the label of the chain is given:

12as, A; 132l, A; 135l, A; 13pk, A; 1a0i, A; 1a0j, A; 1a16, A; 1a26, A; 1a2t, A; 1a41, A; 1a4l, A; 1a4s, A; 1a4y, B; 1a65, A; 1a69, A; 1a79, A; 1a7u, A; 1a8h, A; 1a8s, A; 1a95, A; 1aam, A; 1ab4, A; 1abr, A; 1afr, A; 1afw, A; 1agm, A; 1agy, A; 1aj0, A; 1aj8, A; 1akd, A; 1akm, A; 1ako, A; 1al6, A; 1ald, A; 1alk, A; 1am2, A; 1amo, A; 1amy, A; 1aop, A; 1apx, A; 1aq0, A; 1aq2, A; 1aql, A; 1ar1, A; 1arz, A; 1ast, A; 1asy, A; 1at1, A; 1aug, A; 1aui, A; 1auk, A; 1auo, A; 1ax4, A; 1ay4, A; 1azw, A; 1azy, A; 1b2m, A; 1b2r, A; 1b3r, A; 1b57, A; 1b5d, A; 1b5t, A; 1b65, A; 1b66, A; 1b6b, A; 1b6g, A; 1b6t, A; 1b73, A; 1b7y, A; 1b8f, A; 1b8g, A; 1b93, A; 1b9h, A; 1bbs, A; 1bd3, A; 1be1, A; 1bf2, A; 1bfd, A; 1bg0, A; 1bg6, A; 1bgl, A; 1bhg, A; 1bib, A; 1bix, A; 1bjo, A; 1bjp, B; 1bmt, A; 1bo1, A; 1bol, A; 1boo, A; 1bp2, A; 1bqc, A; 1brm, A; 1brw, A; 1bs0, A; 1bs4, A; 1bs9, A; 1bsj, A; 1btl, A; 1bu7, A; 1bvv, A; 1bvz, A; 1bwd, A; 1bwl, A; 1bwp, A; 1bwz, A; 1bxr, B; 1bya, A; 1bzc, A; 1bzy, A; 1c2t, A; 1c3j, A; 1c4x, A; 1c4z, A; 1c54, A; 1c82, A; 1c9u, A; 1ca2, A; 1cb8, A; 1cbg, A; 1cbx, A; 1cd5, A; 1cde, A; 1cdg, A; 1cel, A; 1cev, A; 1cf2, O; 1cg2, A; 1cg6, A; 1cgk, A; 1chd, A; 1chk, A; 1chm, A; 1cjy, A; 1cm0, A; 1cmx, A; 1cns, A; 1cqg, A; 1ctt, A; 1cv2, A; 1cvr, A; 1cw0, A; 1cwy, A; 1cz0, A; 1cz1, A; 1czf, A; 1d1q, A; 1d2r, A; 1d2t, A; 1d3g, A; 1d4a, A; 1d4c, A; 1d5r, A; 1d6i, B; 1d6o, A; 1d8c, A; 1d8h, A; 1daa, A; 1dak, A; 1db3, A; 1dci, A; 1dco, A; 1dd8, A; 1ddj, A; 1de3, A; 1de6, A; 1dek, A; 1dhf, A; 1dhp, A; 1dhr, A; 1di1, A; 1dii, A; 1dio, A; 1diz, A; 1dj1, A; 1djl, A; 1dki, A; 1dl2, A; 1dli, A; 1dnk, A; 1dnp, A; 1do6, A; 1do8, A; 1dod, A; 1dpg, A; 1ds2, E; 1dub, A; 1dup, A; 1dve, A; 1dwo, A; 1dxe, A; 1dzr, A; 1e0c, A; 1e19, A; 1e1a, A; 1e2t, A; 1e3v, A; 1e6e, A; 1e7q, A; 1e7q, A; 1e94, A; 1eb6, A; 1ebf, A; 1ec9, A; 1ecf, A; 1ecl, A; 1eej, A; 1ef0, A; 1ef8, A; 1eh5, A; 1eh6, A; 1ei5, A; 1els, A; 1emd, A; 1eq2, A; 1esc, A; 1et0, A; 1eu1, A; 1eug, A; 1euu, A; 1euy, A; 1evy, A; 1ex1, A; 1exn, A; 1exp, A; 1eyi, A; 1eyp, A; 1ez1, A; 1ez2, A; 1f2d, A; 1f48, A; 1f6d, A; 1f75, A; 1f7l, A; 1f7u, A; 1f8m, A; 1f8r, A; 1f8x, A; 1fc4, A; 1fcb, A; 1fcq, A; 1fgh, A; 1fgj, A; 1fhl, A; 1fiq, C; 1fnb, A; 1fo6, A; 1fob, A; 1foh, A; 1fps, A; 1fq0, A; 1fr8, A; 1fro, A; 1fui, A; 1fva, A; 1fy2, A; 1g0d, A; 1g64, A; 1g6t, A; 1g72, A; 1g99, A; 1ga8, A; 1gcb, A; 1gcu, A; 1gdh, A; 1ge7, A; 1geq, A; 1gim, A; 1glo, A; 1gog, A; 1gp1, B; 1gpa, A; 1gpj, A; 1gpr, A; 1gq8, A; 1grc, A; 1gsa, A; 1gt7, A; 1gtp, A; 1gxs, A; 1h19, A; 1h3i, A; 1h4g, A; 1h7a, A; 1h7o, A; 1hdh, B; 1hfe, L; 1hka, A; 1hpl, A; 1hqc, A; 1hrd, A; 1hrk, A; 1hti, A; 1hto, A; 1hy3, A; 1hzd, A; 1hzf, A; 1i19, A; 1i1e, A; 1i1i, P; 1i6p, A; 1i78, A; 1i8d, A; 1i8t, A; 1i9a, A; 1idj, A; 1idt, A; 1iec, B; 1ig8, A; 1im5, A; 1ima, A; 1iph, A; 1ir3, A; 1it4, A; 1itq, A; 1itx, A; 1iu4, A; 1j2u, A; 1j49, A; 1j53, A; 1j70, A; 1j7g, A; 1jfl, A; 1jh6, A; 1jhf, A; 1jm6, A; 1jnr, C; 1joa, A; 1jof, A; 1jrp, B; 1js4, A; 1jxh, A; 1k30, A; 1k32, A; 1k4l, A; 1kae, A; 1kas, A; 1kc7, A; 1kcz, A; 1kdg, A; 1kez, A; 1kfu, L; 1kfx, L; 1kim, A; 1knp, A; 1kny, A; 1kp2, A; 1kra, C; 1kzh, A; 1kzl, A; 1l0o, A; 1l1d, A; 1l1l, A; 1l1r, A; 1l6p, A; 1l7d, B; 1l7n, A; 1l7q, A; 1l8t, A; 1l9x, A; 1lam, A; 1lba, A; 1lbu, A; 1lci, A; 1lij, A; 1lio, A; 1ljl, A; 1ltq, A; 1luc, A; 1lvh, B; 1lxa, A; 1lz1, A; 1m21, A; 1m9c, A; 1mbb, A; 1mdr, A; 1mek, A; 1mfp, A; 1mhy, D; 1mj9, A; 1mla, A; 1mok, A; 1moq, A; 1mpx, A; 1mpy, A; 1mqw, A; 1mrq, A; 1mt5, A; 1muc, A; 1mud, A; 1mvn, A; 1myr, A; 1n29, A; 1n2c, C; 1n2c, D; 1n2c, A; 1n2c, F; 1n2c, E; 1n2t, A; 1n5w, B; 1naa, A; 1nba, A; 1nbf, A; 1ndi, A; 1nhx, A; 1nid, A; 1nir, A; 1nkk, A; 1nln, A; 1nlu, A; 1nmw, A; 1nn4, A; 1nsf, A; 1nsj, A; 1nu3, A; 1nvm, A; 1nw9, B; 1nww, A; 1nzy, A; 1o04, A; 1o8a, A; 1o98, A; 1oas, A; 1oba, A; 1odt, C; 1ofd, A; 1ofg, A; 1og1, A; 1ogo, X; 1oh9, A; 1ohh, A; 1ohv, A; 1oj4, A; 1ok4, A; 1onr, A; 1or8, A; 1ord, A; 1otg, A; 1oxa, A; 1oya, A; 1oyg, A; 1p1x, A; 1p4n, A; 1p7m, A; 1pa9, A; 1pad, A; 1pae, X; 1pbg, A; 1peg, A; 1pfk, A; 1pfq, A; 1pgs, A; 1pix, A; 1pj5, A; 1pjb, A; 1pjh, A; 1pkn, A; 1pma, B; 1pma, A; 1pmi, A; 1pnl, B; 1pnt, A; 1pow, A; 1pp4, A; 1ps1, A; 1ps9, A; 1psd, A; 1ptd, A; 1pvd, A; 1pwv, A; 1pxv, A; 1pyl, A; 1pym, A; 1q3q, A; 1q6l,

B; 1q6l, A; 1q6x, A; 1qam, A; 1qaz, A; 1qb4, A; 1qba, A; 1qcn, A; 1qe3, A; 1qfe, A; 1qfl, B; 1qfm, A; 1qfn, A; 1qgn, A; 1qgx, A; 1qh9, A; 1qhf, A; 1qhg, A; 1qho, A; 1qi9, A; 1qj4, A; 1qk2, A; 1qlh, A; 1qq5, A; 1qrg, A; 1qrr, A; 1qrz, A; 1qsg, A; 1qtn, A; 1qv0, A; 1qwn, A; 1qx3, A; 1qz9, A; 1r16, A; 1r1j, A; 1r30, A; 1r44, A; 1r4f, A; 1r4z, A; 1r51, A; 1r6w, A; 1r76, A; 1ra0, A; 1ra2, A; 1rba, A; 1rbl, A; 1rbn, A; 1req, A; 1rhc, A; 1rk2, A; 1rne, A; 1ro7, A; 1roz, A; 1rpt, A; 1rpx, A; 1rql, A; 1rtf, B; 1rtu, A; 1ru4, A; 1s2k, A; 1s3i, A; 1s95, A; 1sca, A; 1ses, A; 1sll, A; 1sme, A; 1smn, A; 1snn, A; 1snz, A; 1sox, A; 1ssx, A; 1stc, E; 1std, A; 1szd, A; 1szj, G; 1t0u, A; 1t7d, A; 1tah, A; 1tde, A; 1tdj, A; 1teh, A; 1thg, A; 1tht, A; 1ti6, A; 1tlp, E; 1tml, A; 1tph, 1; 1trk, A; 1tyf, A; 1tys, A; 1uae, A; 1uag, A; 1uaq, A; 1uas, A; 1uch, A; 1uf7, A; 1ujn, B; 1uk7, A; 1ula, A; 1un1, A; 1uok, A; 1uqr, A; 1uqt, A; 1uro, A; 1ush, A; 1uw8, A; 1v04, A; 1v0e, A; 1v0y, A; 1v25, A; 1vao, A; 1vas, A; 1vid, A; 1vie, A; 1vnc, A; 1vq1, A; 1vr7, A; 1vzx, A; 1vzz, B; 1w0h, A; 1w1o, A; 1wd8, A; 1wnw, A; 1x7d, A; 1x9h, A; 1x9y, A; 1xff, A; 1xqd, A; 1xs1, B; 1xs1, A; 1xtc, A; 1xva, A; 1xx2, A; 1xyz, A; 1y9m, A; 1ybv, A; 1ycf, A; 1ylu, A; 1ysc, A; 1ytw, A; 1z9h, A; 1ze1, A; 1zio, A; 1zym, A; 206l, A; 2a0n, A; 2a86, A; 2aat, A; 2abk, A; 2ace, A; 2acu, A; 2acy, A; 2adm, A; 2alr, A; 2amg, A; 2apr, A; 2ayh, A; 2bbk, M; 2bkr, A; 2bmi, A; 2cnd, A; 2cpo, A; 2cpu, A; 2dhn, A; 2dln, A; 2dor, A; 2dw7, A; 2ebn, A; 2eng, A; 2eql, A; 2f61, A; 2f9z, C; 2gb0, A; 2gsa, A; 2hdh, A; 2hgs, A; 2hi7, B; 2isd, A; 2jcw, A; 2jxr, A; 2lip, A; 2lpr, A; 2nac, A; 2nmt, A; 2oat, A; 2ocp, A; 2pec, A; 2pfl, A; 2pgd, A; 2phk, A; 2plc, A; 2pth, A; 2rnf, A; 2tdt, A; 2thi, A; 2tmd, A; 2toh, A; 2tpl, B; 2tps, A; 2xis, A; 2ypn, A; 3cla, A; 3csm, A; 3eca, A; 3nos, A; 3pca, M; 3pva, A; 3r1r, A; 4kbp, A; 4mdh, A; 5cox, A; 5cpa, A; 5eat, A; 5enl, A; 5fit, A; 5rsa, A; 7atj, A; 7nn9, A; 7odc, A; 8pch, A; 8tln, E;
